# Tanshinones Inhibit the Growth of Breast Cancer Cells through Epigenetic Modification of Aurora A Expression and Function

**DOI:** 10.1371/journal.pone.0033656

**Published:** 2012-04-02

**Authors:** Yi Gong, Yanli Li, Hamid M. Abdolmaleky, Linglin Li, Jin-Rong Zhou

**Affiliations:** 1 Nutrition/Metabolism Laboratory, Department of Surgery, Beth Israel Deaconess Medical Center, Harvard Medical School, Boston, Massachusetts, United States of America; 2 Institute of Molecular and Experimental Therapeutics, East China Normal University, Shanghai, China; Wayne State University School of Medicine, United States of America

## Abstract

The objectives of this study were to evaluate the effects of tanshinones from a Chinese herb *Salvia Miltiorrhiza* on the growth of breast cancer cells, and to elucidate cellular and molecular mechanisms of action. Tanshinones showed the dose-dependent effect on the growth inhibition of breast cancer cells in vitro, with tanshinone I (T1) the most potent agent. T1 was also the only tanshinone to have potent activity in inhibiting the growth of the triple-negative breast cancer cell line MDA-MB231. T1 caused cell cycle arrests of both estrogen-dependent and estrogen-independent cell lines associated with alterations of cyclinD, CDK4 and cyclinB, and induced breast cancer cell apoptosis associated with upregulation of c-PARP and downregulation of survivin and Aurora A. Among these associated biomarkers, Aurora A showed the most consistent pattern with the anti-growth activity of tanshinones. Overexpression of Aurora A was also verified in breast tumors. The gene function assay showed that knockdown of Aurora A by siRNA dramatically reduced the growth-inhibition and apoptosis-induction activities of T1, suggesting Aurora A as an important functional target of T1 action. On the other hand, tanshinones had much less adverse effects on normal mammary epithelial cells. Epigenetic mechanism studies showed that overexpression of Aurora A gene in breast cancer cells was not regulated by gene promoter DNA methylation, but by histone acetylation. T1 treatment significantly reduced acetylation levels of histone H3 associated with Aurora A gene. Our results supported the potent activity of T1 in inhibiting the growth of breast cancer cells in vitro in part by downregulation of Aurora A gene function. Our previous studies also demonstrated that T1 had potent anti-angiogenesis activity and minimal side effects in vivo. Altogether, this study warrants further investigation to develop T1 as an effective and safe agent for the therapy and prevention of breast cancer.

## Introduction

Breast cancer is the most common form of cancer in women and the leading cause of cancer death in American women with over 207,090 new cases of invasive breast cancer in women and about 39,840 deaths from breast cancer in 2010 [1]. Current therapies for breast cancer usually have variable effectiveness with high toxicity to normal tissues, and breast tumors often develop metastasis and drug resistance. Therefore, searching for effective regimens with minimal side effects remains the top priority in breast cancer research.

Danshen (*Salvia miltiorrhiza Bunge*) has been widely used in traditional Chinese medicine practice for centuries in the treatment of coronary artery disease and cerebrovascular diseases with minimal side effects. Cryptotanshinone (CT), tanshinone IIA (T2A) and tanshinone I (T1) are three major diterpene compounds of tanshinones in Danshen. In addition to their functions in cardiovascular systems, tanshinones have been recently shown to possess some activities against human cancer cells. CT inhibited the growth of hepatocarcinoma cells [2] in vitro via cell cycle arrest at S phase and the growth of gastric and hepatocellular cancer cells. T2A inhibited the growth of breast cancer [3], [4], nasopharyngeal carcinoma [5], glioma [6], leukemia [7] and hepatocellular carcinoma [8], [9] cells in vitro by induction of apoptosis [5], [8]. T2A also inhibited invasion of lung cancer cells in vitro [10]. T1 inhibited the growth of leukemia [11], lung [12] and breast cancer [13], [14] in vitro in part via induction of apoptosis. However, the relative activity of tanshinones against breast cancer is unclear, and their functional targets and molecular mechanisms remain elusive.

The objectives of this study were to evaluate the activity of tanshinones in inhibiting the growth of breast cancer cells, to identify functional targets of tanshinones, and to understand the epigenetic mechanisms by which tanshinones regulate the expression of functional targets.

## Results

### Effects of Tanshinones on Cell Growth of Breast Cancer Cell Lines and HMEC

**Figure 1:**
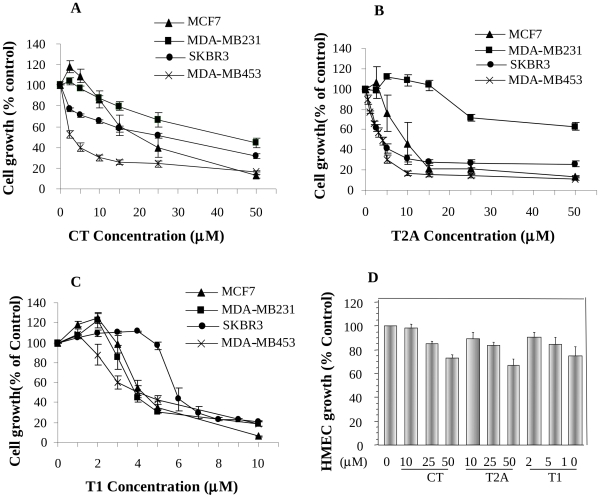
The dose-dependent effects of CT (**A**)**, T2A** (**B**) **and T1** (**C**) **on the growth of human breast cancer cell lines (MCF-7, MDA-MB231, SKBR3 and MDA-MB453) and on normal mammary epithelial cells (HMEC)** (**D**)**.** Values were mean±SEM of at least three independent experiments, each in triplicates.

As shown in Fig. 1, tanshinones inhibited the growth of breast cancer cells in both dose- and cell line-dependent manners. CT inhibited cell growth of different breast cancer cell lines with IC_50_ between 5–50µM; among four cell lines, MDA-MB453 was the most sensitive one with IC_50_ around 5µM, while MDA-MB231 was the least sensitive one with IC_50_ around 50µM (Fig. 1A). T2A inhibited the growth of breast cancer cell lines with MDA-MB-453 the most sensitive one (IC_50_ = 3.5µM) and MDA-MB-231 the least sensitive one (IC_50_ >50µM) (Fig. 1B). On the other hand, T1 showed the potent activity in inhibiting the growth of all breast cancer cell lines with IC_50_′s between 4–9µM (Fig. 1C). Generally, among three tanshinones, CT showed less activity, T1 and T2A showed similar activities in inhibiting the growth of MDA-MB453 and SKBR3 cell lines, but T1 was more potent than T2A in inhibiting the growth of MCF-7 and especially MDA-MB231 cell lines. On the other hand, tanshinones showed much less cytotoxicity on normal mammary epithelial cells (HMEC) (Fig. 1D). The results suggest that tanshinones may have potent anti-growth effects on breast cancer cells, but limited adverse effect on normal cells.

### Effects of T1 on Cell Cycle Progression of Breast Cancer Cells and Modulation of Related Molecular Markers

**Figure 2:**
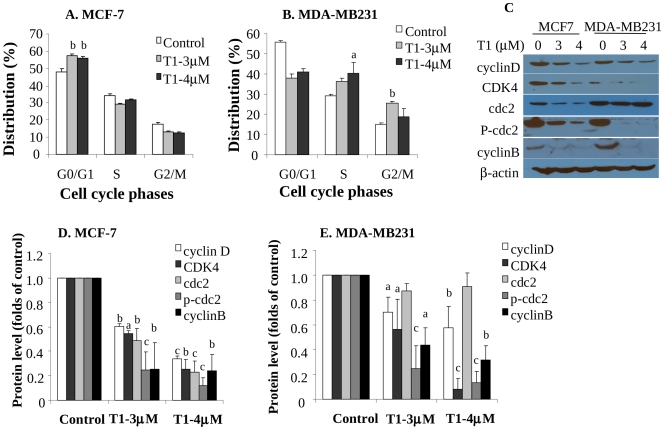
Effects of T1 on Cell Cycle Progression and Protein Levels of Cell Cycle-Related Biomarkers (48h). A and B: Effects of T1 on cell cycle arrests of estrogen-dependent MCF-7 (A) and estrogen-independent MDA-MB231 (B) cell lines. Data were from at least two independent experiments, each in duplicates; C: The representative Western blot images showing the effects of T1 on protein levels of cell cycle related biomarkers cyclinD, CDK4, cdc2, p-cdc2 and cyclinB; D and E: Quantitation of cyclinD, CDK4, cdc2, p-cdc2 and cyclinB protein levels in MCF-7 (D) and MDA-MB231 (E) by densitometry after normalization to β-actin. Values were mean±SEM of at least two independent experiments. Within the panel, the value with a letter was significantly different from that of the corresponding control, a, p<0.05; b, p<0.01; c, p<0.001.

Since T1 showed the potent effect on all breast cancer cell lines, in the following studies, we mainly focused on T1 to determine its cellular and molecular mechanisms in both estrogen-dependent MCF-7 and estrogen independent MDA-MB231 cell lines. The cell cycle analysis data showed that T1 caused a G_0_/G_1_ phase arrest in MCF-7 (Fig. 2A, P<0.01) and both S and G_2_/M phase arrests in MDA-MB231 (Fig. 2B, P<0.05). The representative FACS histograms are shown as Fig. S1.

In order to examine molecular alterations associated with cell cycle arrest, we determined protein expression levels of several cell cycle related markers. T1 treatment significantly down-regulated cyclin D, CDK4 and cyclin B protein levels in both MCF-7 (Fig. 2C and 2D) and MDA-MB231 cell lines (Fig. 2C and 2E). T1 also significantly downregulated protein levels of cdc2 and its active form, phosphorylated cdc2 (p-cdc2) in MCF-7 cell line (Fig. 2C and 2D) and p-cdc2 level in MDA-MB231 cell line (Fig. 2C and 2E).

### Effects of T1 on Apoptosis of Breast Cancer Cells and Modulation of Related Molecular Markers

**Figure 3:**
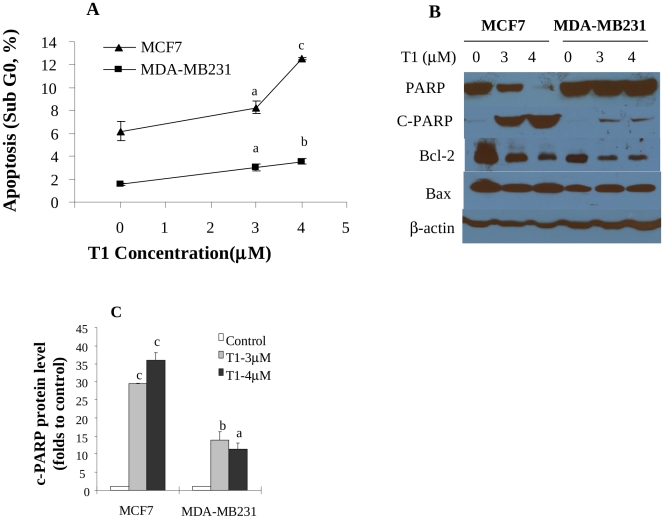
Effects of T1 on apoptosis of breast cancer cells and protein levels of apoptosis-related biomarkers (48h). A: Effects of T1 on the proportion of DNA fragmentation (sub-G_0_), a marker of apoptosis, in MCF-7 and MDA-MB231 cell lines. Values were mean±SEM of at least two independent experiments, each in duplicates; B: The representative Western blot images showing the effects of T1 on protein levels of apoptosis related biomarkers PARP, c-PARP, bcl2 and bax; C: Quantitation of c-PARP protein levels in MCF-7 and MDA-MB231 by densitometry after normalization to β-actin. The images for quantitation were from at least two independent experiments. Within the panel, the value with a letter was significantly different from that of the corresponding control, a, p<0.05; b, p<0.01; c, p<0.001.

Aside from the disturbance of cell cycle, T1 was also found to induce apoptosis in breast cancer cells. T1 treatment increased the proportion of Sub-G_0_ cells, a parameter of apoptosis, in both MCF-7 and MDA-MB231 cell lines in a dose-dependent manner (Fig. 3A). T1 at 3 and 4µM significantly increased the proportion of Sub-G_0_ MCF-7 cells to 8% (1.3 folds, P<0.05) and 12% (2.0 folds, P<0.005), respectively, compared with 6% in the control (Fig. 3A). Similarly, T1 (3 and 4µM) increased the proportion of Sub-G_0_ MDA-MB231 cells to 3% (2.0 folds, P<0.05) and 3.5% (2.3 folds, P<0.01), respectively, compared with 1.5% in the control (Fig. 3A).

Consistent with cellular results, T1 treatment significantly increased the protein level of an important apoptosis related protein marker, cleaved PARP (c-PARP) in both MCF-7 and MDA-MB231 cells (Fig. 3B and 3C, P at least <0.05). Other apoptosis related markers, bcl-2 and bax were also examined, and the results showed that T1 reduced bcl-2 protein levels in both MCF-7 and MDA-MB231 cell lines, but had no effect on bax levels (Fig. 3B).

### Gene Expression and Protein Levels of Survivin and Aurora A levels in Human Breast Tumors and Breast Cancer Cell Lines

**Figure 4:**
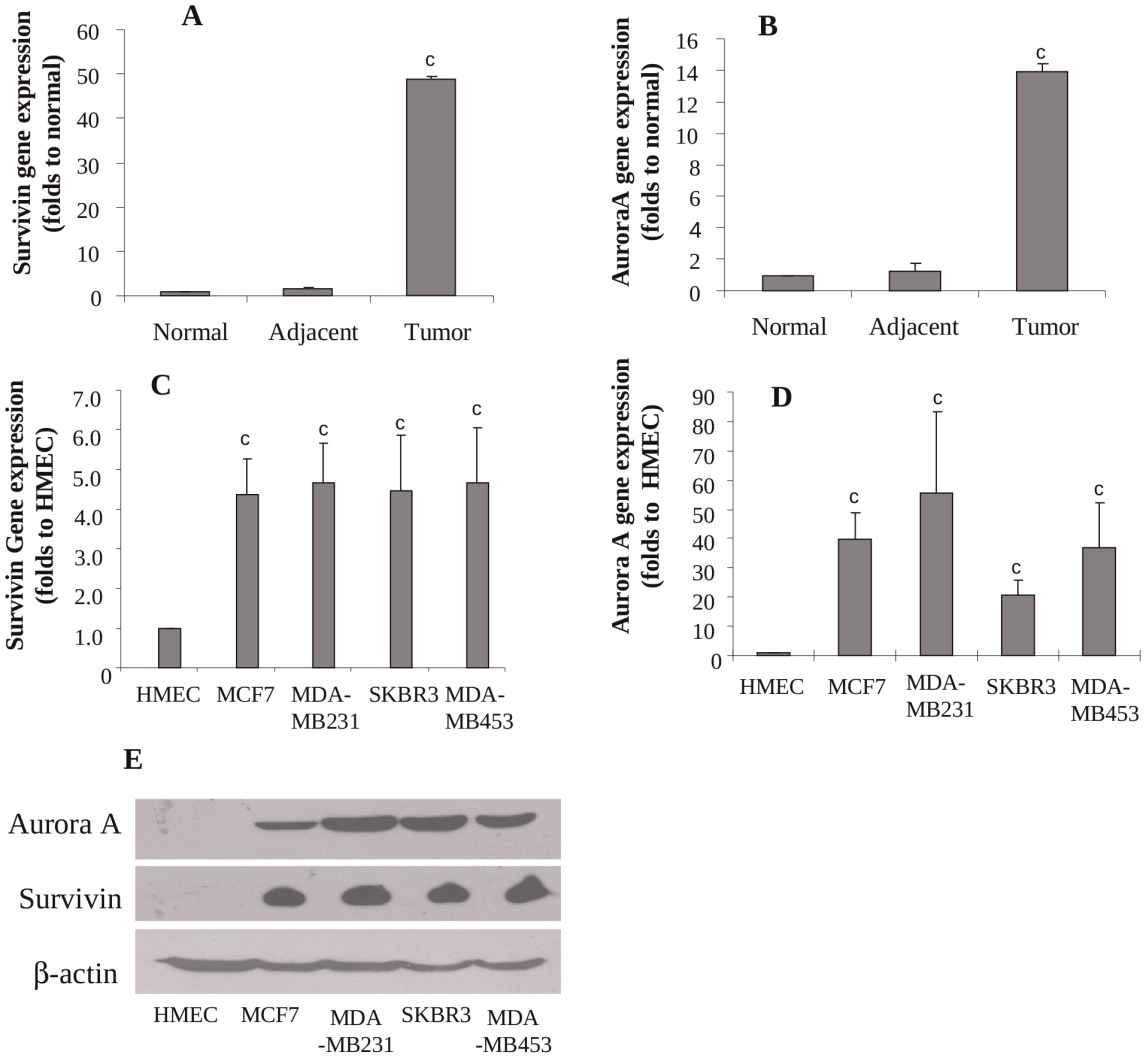
Expressions of survivin and Aurora A in human breast tissues and breast cancer cells. A and B: Expressions of survivin (A) and Aurora A (B) genes in normal breast tissues (n = 10), normal tissues adjacent to tumors (n = 12) and breast tumors (n = 14) by real-time RT-PCR; C and D: Expressions of survivin (C) and Aurora A (D) genes in human breast cancer cell lines (MCF-7, MDA-MB231, SKBR3 and MDA-MB453) and HMEC; E, Protein levels of survivin and Aurora A in HMEC and human breast cancer cell lines by western blot. Values were mean±SEM. Within the panel, the value with a letter was significantly different from that of the corresponding control, c, p<0.001.

In addition to above molecular markers, we further identified other molecular markers that might be responsive to and responsible for the T1 activity. In this study, survivin and Aurora A were investigated as putative targets of T1 mechanism of action, because these biomarkers were differentially expressed in cancer cell lines in comparison to normal mammary epithelial cells. Survivin (Fig. 4C) and Aurora A (Fig. 4D) genes were significantly upregulated in human breast cancer cell lines by 4–5 folds and 20–50 folds respectively, compared with that in HMEC. Western blot analysis confirmed overexpression of survivin and Aurora A protein levels in breast cancer cell lines (Fig. 4E). We further compared the expression of survivin and Aurora A genes among human breast tumors, breast tissues adjacent to breast tumors and breast tissues from healthy subjects. The results showed that both survivin and Aurora A genes were extremely low in both normal breast tissues of healthy women and breast tissues adjacent to breast tumors of breast cancer patients, but were dramatically elevated in breast tumor tissues by about 49 (Fig. 4A) and 14 folds (Fig. 4B), respectively. Due to limited amount of tissues, protein levels were not measured in human breast tissue samples.

### Effects of Tanshinones on the Expression of Survivin and Aurora A in Breast Cancer Cells

**Figure 5:**
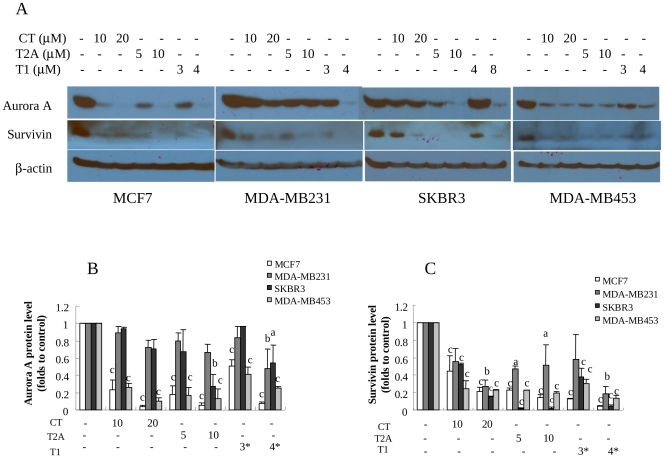
Effects of tanshinones on survivin and Aurora A protein levels in breast cancer cells (48 h). A: Representative western blot images showing survivin and Aurora A protein levels in breast cancer cell lines (MCF-7, MDA-MB231, SKBR3, MDA-MB453) following tanshinone treatments with β-actin as the loading control; B and C: Quantitation of Aurora A (B) and survivin (C) protein levels by densitometry after normalization to β-actin. Values were mean±SEM of three independent experiments in duplicates. The images for quantitation were from at least two independent experiments. Within the panel, the value with a letter was significantly different from that of the corresponding control, a, p<0.05; b, p<0.01; c, p<0.001.

We further measured the effects of tanshinones on the survivin and Aurora A protein levels to determine if Aurora A and/or survivin are/is the functional molecular targets for tanshinones. As shown in Fig. 5 (A and B), Aurora A protein levels were downregulated by tanshinones in a dose-dependent manner. It is very important to note that the Aurora A protein level in SKBR3 cell line, the least sensitive one to T1 treatment, was not downregulated by T1 at 4µM, but significantly downregulated by T1 at 8µM, which was consistent with the T1 activity in cell growth inhibition. Similarly, MDA-MD231 cell line was the least sensitive one to CT and T2A, and Aurora A protein levels were not downregulated by CT or T2A treatment, but significantly downregulated by T1 treatment (at 3 and 4****µM) (Fig. 5A and 5B). These results strongly support the correlation between cell growth-inhibition activities of tanshinones and downregulation of Aurora A protein levels and thus suggest that Aurora A may be an important functional target of tanshinones. Further experimental results also showed that Aurora A gene expression, in parallel with Aurora A protein level, was significantly downregulated by T1 treatment in MCF-7 and MDA-MB231 cell lines (Fig. S2).

On the other hand, despite different sensitivities of breast cancer cell lines to tanshinones, survivin was universally downregulated by tanshinones (Fig. 5A and 5C). These results suggest that survivin may not be the direct molecular target of tanshinones.

### The Effect of Aurora A Silencing on T1 Activity

**Figure 6:**
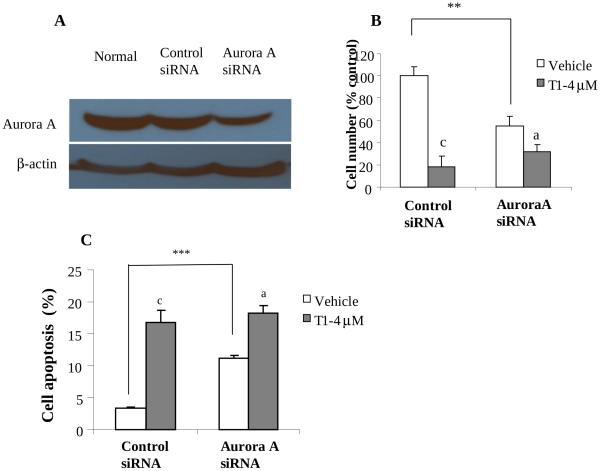
Effects of Aurora A knockdown on the T1 activities in growth and apoptosis of MCF-7 breast cancer cells. A: Western blot analysis showing knockdown of Aurora A protein level in MCF-7 cells by Aurora A siRNA; B: Effect of Aurora A knockdown on the growth-inhibition activity of T1; C: Effect of Aurora A knockdown on the apoptosis-induction activity of T1. Values were mean±SEM of three independent experiments in duplicates. Within the panel, the value with a letter was significantly different from that of the corresponding control (c, p<0.001), and the values with a “*” are significantly different (**, P<0.01; ***, P<0.001).

To further determine if Aurora A is a functional target of T1 actions in inhibiting the growth and inducing apoptosis of breast cancer cells, we used Aurora A specific siRNA to inhibit the expression of Aurora A in MCF-7 cells and measured the effect of Aurora A knockdown on T1 activity. The siRNA inhibited Aurora A gene expression in a dose-dependent manner. To sensitively evaluate the effect of siRNA on T1 activity, we purposely used a dose of siRNA that downregulated Aurora A protein level in MCF-7 cells by 46% (Fig. 6A) and in parallel significantly reduced cell growth by 45% (Fig. 6B, P<0.001). Aurora A knockdown reduced T1 activity in inhibiting the growth of MCF-7 cells. T1 at 4µM inhibited the growth of the vector-control MCF-7 cells by 80% (P<0.001), but inhibited the growth of Aurora A-knockdown MCF-7 cells by 40% (P>0.05) (Fig. 6B), indicating that Aurora A knockdown (by 46%) reduced the T1 activity by 50% (from 80% inhibition to 40% inhibition). Additionally, we determined the effect of Aurora A knockdown on the apoptosis-induction activity of T1. Aurora A knockdown (by 46%) significantly increased MCF7 cell apoptosis by 3 folds (Fig. 6C, P<0.001); T1 (4****µM) significantly induced apoptosis of the control-siRNA MCF-7 cells by around 5 folds, but it induced apoptosis of the Aurora A-knockdown cells by 50% only (Fig. 6C). These results suggest Aurora A as an important functional target of T1 action.

### Epigenetic Modifications of Aurora A Expression in Breast Cancer Cells

**Figure 7:**
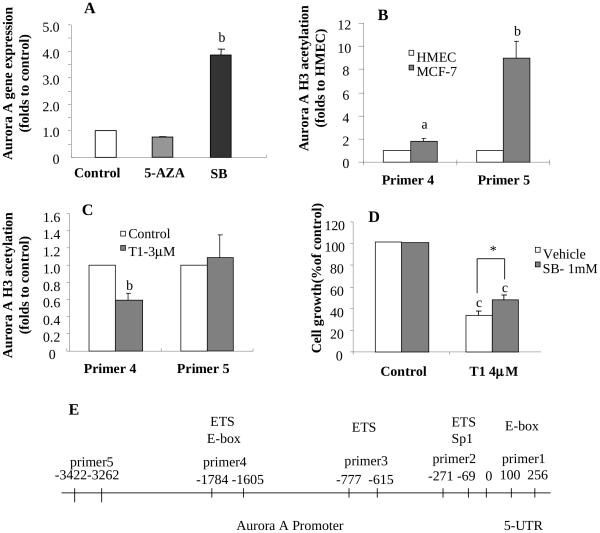
Epigenetic modifications of Aurora A expression by T1 treatment in breast cancer cells. A: Effect of the demethylating agent 5′-Azacytine (5-AZA) or the histone deacetylase inhibitor sodium butyrate (SB) treatment on Aurora A gene expression in MCF-7 cells; B: Identification of histone H3 acetylation level of DNA promoter areas in Aurora A gene that are associated with overexpression of Aurora A gene in MCF-7 cells by CHIP; C: Effects of T1 treatment (3****µM) on acetylation levels of histone 3 of Aurora A gene by CHIP; D: Effects of SB (1 mM) treatment on the activity of T1 in inhibiting the growth of MCF-7 cells; E: Scheme showing the CHIP primer locations for Aurora A gene. Values were mean±SEM of three independent experiments in triplicates. Within the panel, the value with a letter is significantly different from that of the corresponding control (a, p<0.05; b, p<0.01), and the values with a “*” are significantly different (*, P<0.05).

We further investigated possible epigenetic mechanism(s) that might be responsible for Aurora A overexpression in breast cancer cells and for explaining the T1 activity in downregulating Aurora A expression associated with growth inhibition of breast cancer cells. When MCF-7 cells were treated with 5-AZA, a DNA demethylating regent, Aurora A gene expression was not altered (Fig. 7A), suggesting that Aurora A gene expression in breast cancer cells may not be regulated by DNA methylation. The raw Ct values are listed in Table S2. Further MSP analysis showed that Aurora A gene DNA promoter had limited degree of methylation (Fig. S3). Bisulfite-treated DNA sequencing also confirmed that Aurora A gene promoter was primarily unmethylated (data not shown).

On the other hand, breast cancer cells (MCF-7) treated with sodium butyrate (SB), a histone deacetylase inhibitor, had an increased level of Aurora A gene expression by 4 folds (Fig. 7A), suggesting that Aurora A gene expression in breast cancer cells may be regulated, at least in part, by histone acetylation. The raw Ct values are listed in Table S2.

We further examined the histone H3 acetylation levels in HMEC and MCF-7 cells using CHIP Q-PCR assay. Five pairs of primers (Table S1) were used for detecting possibly altered sites in the Aurora A promoter region. The locations covered by these paired primers are shown in Fig. 7E. No obvious changes of Aurora A H3 histone acetylation were found in the areas where primer 1, primer 2 or primer 3 amplified. On the other hand, the areas where primer 4 and primer 5 amplified had increased H3 acetylation levels by 1.8 (P<0.05) and 8.9 (P<0.01) folds, respectively, in MCF-7 cells (Fig. 7B). The raw Ct values are listed in Table S2.

### Effect of T1 on Alteration of Histone Acetylation Associated with Aurora A Gene DNA Promoter

We further determined if T1 downregulated Aurora A gene expression in part via alteration of histone acetylation in Aurora A gene DNA promoter. T1 treatment significantly decreased H3 acetylation level in primer 4-amplified area by over 40% (P<0.05), but it didn’t significantly alter H3 acetylation levels in the primer 5-amplified area (Fig. 7C). The results suggest that T1 may downregulate Aurora A gene expression by reducing acetylation of H3 associated with the primer 4-amplified area in Aurora A gene DNA promoter.

We also determined the influence of SB on the T1 activity in inhibiting MCF-7 cell growth. As shown in Fig. 7D, the presence of SB (increased histone acetylation and upregulated Aurora A expression) reduced the growth inhibition activity of T1 by 25% (P<0.05). This suggests that modulation of histone acetylation is an important epigenetic mechanism by which T1 down-regulates the expression and function of Aurora A.

## Discussion

In the present study, we evaluated the activity of a group of natural components, tanshinones (CT, T1 and T2A) from a Chinese herb *Salvia Miltiorrhiza* (Danshen) in inhibiting the growth of human breast cancer cells. Among these compounds, T1 showed the most potent anti-growth activity against both estrogen-dependent and estrogen-independent breast cancer cells via cell cycle arrest and induction of apoptosis. On the other hand, tanshinones showed much less adverse effects on the growth of HMEC. Determination of biomarkers showed that downregulation of Aurora A was correlated to the anti-growth activity of tanshinones. The gene function assay showed that Aurora A knockdown by siRNA reduced the anti-growth and pro-apoptotic activities of T1. Epigenetic mechanism studies showed that overexpression of Aurora A in breast cancer cells was, at least in part, modulated by increased acetylation of histone associated with Aurora A gene promoter, but not altered gene promoter methylation. Further studies showed that T1 significantly decreased histone acetylation level associated with a specific region in Aurora A gene promoter. Our study provided at the first time, to the best of our knowledge, the experimental evidence to suggest T1 as the potent agent in inhibiting the growth of breast cancer cells and Aurora A as an important functional target for T1 action via epigenetic mechanism of histone acetylation.

The Aurora kinases are a novel oncogenic family of mitotic serine/threonine kinases (S/T kinases) that are involved in the processes of cell division [15]. Up till now, three Aurora kinases, A, B and C, have been identified in humans [16], [17], [18]. Among the three kinases, Aurora kinase A is a key kinase that is important in chromosomal distribution. Aurora A is localized on duplicated centrosomes and spindle poles during mitosis and is required for the timely entry into mitosis and proper formation of a bipolar mitotic spindle by regulating centrosome maturation, separation, and microtubule nucleation activity [19]. Aurora A is frequently overexpressed in a number of human cancers, such as bladder [20], [21], breast [22], colon [17], [23], pancreatic [24] and prostate [25], [26], [27], [28], [29] cancers and is recognized as one of the important molecular targets for cancer therapy [30], [31], [32].

In the present study, we, at the first time, demonstrated that the activity of tanshinones in breast cancer cell growth inhibition was primarily due to downregulation of the expression and function of Aurora A. Cautions should be noted that we performed the gene function assay by knocking down Aurora A gene expression only, but did not perform the Aurora A overexpression assay. This is the limitation of the current study, and more experiments using Aurora A overexpression assay to determine if Aurora A overexpression could rescue prostate cancer cells from apoptosis induced by T1 would provide another line of important evidence to suggest tanshinones as a novel group of Aurora A inhibitors. Our previous studies also showed that T1 also had potent anti-angiogenesis activity and inhibited the growth of prostate cancer in vitro and in vivo [33], but with minimal side effect on food intake and body weight. These results provide important scientific evidence to support further investigations to develop tanshinones, especially T1 as effective therapeutic agents against breast cancer.

It is becoming widely accepted that epigenetic alterations are universally present in human malignancies. Epigenetic alterations of the genome such as DNA promoter methylation and chromatin remodeling play an important role in tumorigenesis [34], [35]. Recent findings also indicate epigenetic modifications as key factors in breast carcinogenesis, and as important targets for preventative care and therapeutics because of their potential for reversal [36], [37]. Epigenetic modification has been recognized as an important mechanism by which a variety of natural bioactive compounds exert their anti-cancer effect [38], [39], [40], [41]. However, it has not been reported if epigenetic mechanism is responsible for the tanshinones’ anti-cancer activity. Our current study provided, at the first time, the promising evidence to support that histone acetylation is an important epigenetic mechanism that behinds the overexpression of Aurora A in breast cancer and governs the downregulation of Aurora A function by tanshinones. This study also supports future investigation to understand how histone acetylation in the primer 4-amplified region of Aurora A gene promoter significantly modulates Aurora A gene expression.

It is important to note that the triple-negative breast cancer (TNBC) cell line MDA-MB231 was very sensitive to T1, but not CT or T2A. TNBC is clinically characterized as more aggressive and less responsive and more resistant to standard treatment. Searching for effective strategies for the treatment of TNBC has become the top priority in breast cancer therapy. Our results warrant further investigation to determine if T1 may serve as a novel candidate agent for the management of TNBC. Identification of T1 as a potent anti-TNBC agent could have significant impact on developing novel therapeutic strategies for the treatment of TNBC.

Our studies showed that T1 inhibited the growth of breast cancer cell lines at the IC_50_ doses of 4–9µM (Figure 1). Previous studies have indicated that the blood levels of tanshinones after oral administration could reach the high nM range [42], [43], [44], [45]. This may raise the concern if tanshinones may have significant activity in vivo. On the other hand, our previous animal studies showed that T1 had potent in vivo activity in inhibiting the growth of prostate [33] and lung tumors [46]. Although the blood T1 levels were not determined, it would be expected to be below the in vitro IC_50_ levels. We hypothesize that the optimal growth conditions employed in the in vitro studies with carefully controlled media conditions, temperature and oxygenation may not be predictive of the complex and harsh in vivo conditions in the tumor microenvironment, in which hypoxia, necrosis, and suboptimal perfusion and diffusion limit nutrient availability and removal of metabolic waste. It is thus imperative to apply clinically relevant animal models to verify the efficacy of tanshinone treatment at safe doses.

In conclusion, our study provided at the first time, to the best of our knowledge, the supporting evidence to suggest that T1 have potent anti-breast cancer activity in part via downregulation of Aurora A expression and function. Our results also suggest further research in developing tanshinones and their derivatives as novel Aurora A-targeting drug candidates. Moreover, our results warrant further investigation to evaluate the effect of T1 on TNBC. Together with our previous findings that T1 also had potent anti-angiogenesis activity and minimal side effects in vivo, our studies provide strong evidence to support further investigations on developing T1 as effective and safe agent for the therapy and prevention of breast cancer.

## Materials and Methods

### Ethics Statement

Breast tumors and breast tissues adjacent to tumors from breast cancer patients and normal breast tissues from healthy women were purchased from Cooperative Human Tissue Network (Philadelphia, PA). The protocol was reviewed and approved by the Committee on Clinical Investigation at Beth Israel Deaconess Medical Center.

### Materials

Tanshinones (CT, T2A and T1) were purchased from LKT (St. Paul, MN), and the purity was verified by high performance liquid chromatography. Tissue culture media, fetal bovine serum (FBS), and trypsin were purchased from Life Technologies, Inc. (Grand Island, NY). 5′-Azacytidin (5-AZA), Sodium butyrate (SB) and Propidium iodide (PI) were from Sigma (St. Louis, MO). RNase A and 3-(4,5-dimethyl-thiazol-2yl)-5-(3-carboxymethoxyphenyl) -2- (4-sulfophenyl)-2H-tetrazolium (MTS) were from Promega (Madison, WI).

The antibodies used in this study were: cyclin B1 (Oncogene Research Products, Boston, MA), Bcl-2 (Santa Cruz Biotechnology, Santa Cruz, CA), c-PARP, bax, cyclin D, CDK4, cdc2, p-cdc2, Aurora kinase A and survivin (Cell Signaling, Beverly, CA) and β-actin (Merck Co., Darmstadt, Germany).

### Cell Lines and Human Tissues

Human breast cancer cell lines (MCF-7, MDA-MB231, SKBR3, MDA-MB453) were obtained from American Type Culture Collection and maintained in Dulbecco’s modified Eagle’s medium (DMEM) supplemented with 10% (v/v) heat-inactivated FBS and antibiotics. All cells were maintained at 37^o^C in a humidified atmosphere of 95% air and 5% CO_2_. Human mammary epithelial cells (HMEC) were obtained from Lonza (Walkersville, MD) and cultured in mammary epithelium basal medium (MEBM) plus MEGM single quotes (Lonza) at 37^o^C in a humidified atmosphere of 95% air and 5% CO_2_.

### Cell Growth Assay

The effects of Tanshinones on cell growth were determined by using Cell Titer 96 Aqueous One Solution Reagent, MTS (Promega) as described previously [47]. Briefly, MCF-7 (6000), MDA-MB231 (3000), SKBR3 (7000), or MDA-MB453 (8000) cells were plated in each well of 96-well plate and allowed to attach overnight. Cells were then treated with tanshinones at desired concentrations or dimethyl sulfoxide (DMSO) as the vehicle and incubated for 72 hours. MTS was added and incubated for 2 to 4 hours at 37^o^C in 5% CO_2_ and light absorbance of formazan was measured at 490 nm in a microplate reader (VersaMax, Molecular Device, Sunnyvale, CA). The experiments were independently performed at least three times, each in triplicates. The results were confirmed by direct cell counting using a hemocytometer.

### Cell Cycle Analysis and DNA Fragmentation

Cell cycle distribution and DNA fragmentation as a marker of apoptosis in MCF-7 and MDA-MB231 cells were determined by flow cytometry following the described procedures (Becton Dickinson, Immunocytometry Systems, Mountview, CA) for cell cycle distribution using programs provided by Becton Dickinson. Briefly, cells (MCF-7 and MDA-MB231) were treated with T1 at desired concentrations for 48 hours, collected, fixed and then stained with 50µg/ml PI (together with 50µg/ml RNase A). Stained cells were analyzed by using FACScans (Becton Dickinson, Mountview, CA) for fragmented DNA and cell cycle using programs provided by Becton Dickinson. The Sub-G_0_ proportion represented DNA fragmentation and was considered as a parameter of apoptosis. The experiment was independently performed repeated at least once, each in duplicates.

### Annexin V and PI Staining for Apoptosis Detection

The effect of Aurora A silencing on the apoptosis-induction activity of T1 was determined by Annexin V-PI apoptosis detection kit (Chemicon International Inc, Billerica, MA) following the instruction of kit. Briefly, treated MCF-7 cells were resuspended in Annexin V solution and incubated at room temperature for 15 min, PI was then added for another 5-min incubation in the dark. Apoptotic cells were analyzed by flow cytometry (Becton Dickinson, Immunocytometry Systems, Mountview, CA). The experiments were independently performed at least twice, each in duplicates.

### Quantitative Real Time Reverse Transcription-PCR

Total RNA was isolated by using Qiagen RNeasy Mini Kit (Qiagen, Valencia, CA). First-strand cDNA synthesis used 100ng random primer (Invitrogen, Carlsbad, CA), 1.0µg of total RNA, 10mM dNTP and 200 units of reverse transcriptase (Invitrogen, Carlsbad, CA) per 20µl reaction. The sequences of primers used in this study were listed in Table S1. PCRs were performed in a 25**µ**l final volume by using SYBR Green master mix from SABiosciences (SABiosciences, Frederick, MD). Relative mRNA expression was calculated by the ΔΔCt comparative methods using β-actin as an internal control.

### Aurora A Silencing by siRNA

The Aurora A silencing by siRNA followed the method described by Lentini and coworkers [23] with appropriate modifications. Briefly, 8×10^4^ MCF-7 cells were seeded in a 6 well plate and incubated for 24h. The silencer negative control and siRNA for Aurora A (Ambion, Austin, TX) were diluted in Opti-MEM I Reduced Serum Medium (Invitrogen, Carlsbad, CA) and transfected with Lipofectamine 2000 according to the manufacturer’s instructions. The final concentration of siRNA added to the cells was 33 nM. The duplex siRNA sequence for Aurora A was as follows: 5′-AUGCCCUGUCUUACUGUCATT-3′.

### Sodium Bisulfite Treatment of DNA and Methylation Specific PCR (MSP) Analysis

The DNA promoter methylation status of specific gene was calculated by using sodium bisulfite treatment of DNA and methylation specific PCR (MSP) analysis [48]. Briefly, DNA was extracted by using DNeasy Blood & Tissue Kit (Qiagen, Valencia, CA). 2µg genomic DNA was diluted in 20µL water and subjected to bisulfite DNA conversion according to the protocols provided in the Epitect Bisulfite Kit (Qiagen, Valencia, CA). The converted DNA was then purified for MSP analysis by using PCR amplifications. A typical reaction consisted of 2.5 µL of 10X standard PCR buffer, 0.4 µL 10 µM dNTP, 2.5 U platinum Taq DNA polymerase (Invitrogen), 25 pmol of each methylated or unmethylated DNA-specific primers, and ultrapure DNAse-/RNAse-free water in a total volume of 25 µL. PCR products will be resolved on non-denaturing polyacrylamide gels, stained with ethidium bromide, and visualized under UV illumination.

### Chromatin Immunoprecipitation (CHIP)

The experiment was performed according to the description of the Magna ChIP^TM^& EZ-Magna ChIP^TM^ Kit (Millipore, Billerica, MA). In brief, cells were fixed by formaldehyde and harvested. The cells were then broken open for nuclear extraction and the DNA was sheared to 200–1000bp fragments by sonication. The sonicated DNA was used for immunoprecipitation by using anti-acetyl histone antibody. The immunoselected DNA was further purified and analyzed by quantitative real-time PCR using specific primers of Aurora A (Table S1). Q-PCR values were normalized to input and enrichment compared to IgG control samples.

### Western Blot Analysis

Cells were treated with different concentrations of tanshinones, cell lysates were prepared, and protein expression was determined following the procedures we previously described [47], [49]. The protein levels were quantitated by using densitometric analysis by using NIH image analysis software and expressed as percentages of the control after being normalized with the housekeeping protein of β-actin. The primary antibodies used were Aurora A (1∶1000), bcl-2 (1∶200), cyclin B (1∶1000), cdc2 (1∶1000), CDK4 (1∶1000), cyclin D (1∶1000), c-PARP (1∶1000), p-cdc2 (1∶1000), survivin (1∶1000) and β-actin (1∶10,000).

### Statistical Analysis

Results were expressed as group means±SEM and analyzed for statistical significance by analysis of variance followed by Fisher’s protected least-significant difference based on two-side comparisons among experimental groups by using Statview 5.0 program (SAS Institute, Inc., Cary, NC). A *P* < 0.05 was considered statistically significant.

## Supporting Information

Figure S1 **Representative FACS histograms showing the effects of T1 treatments (3 and 4**µ**M) on cell cycle progression in MCF-7** (**A-C**) **and MDA-MB231** (**D-F**) **cell lines.**
(TIF)

Figure S2 **Effects of T1 treatment (4**µ**M) on Aurora A gene expression in MCF-7 cells.** Values were mean±SEM of three independent experiments in triplicates. The value with a letter is significantly different from that of the corresponding control (c, p<0.001).
(TIF)

Figure S3 **The representative image showing unmethylation status of Aurora A gene DNA promoter in MCF-7, MDA-MB231, SKBR3 and MDA-MB453 human breast cancer cell lines, as determined by methylation specific PCR (MSP).**
(TIF)

Table S1 **Sequences of the primers.**
(DOC)

Table S2 **Raw Ct values for** **Figure 7** **A, B, and C.**
(DOC)
