# Supplementary figures and images for: Tanshinones Inhibit the Growth of Breast Cancer Cells through Epigenetic Modification of Aurora A Expression and Function
Source: PLoS One. 2012 Apr 2;7(4):e33656. doi: 10.1371/journal.pone.0033656 (PMC3317444; doi:10.1371/journal.pone.0033656)

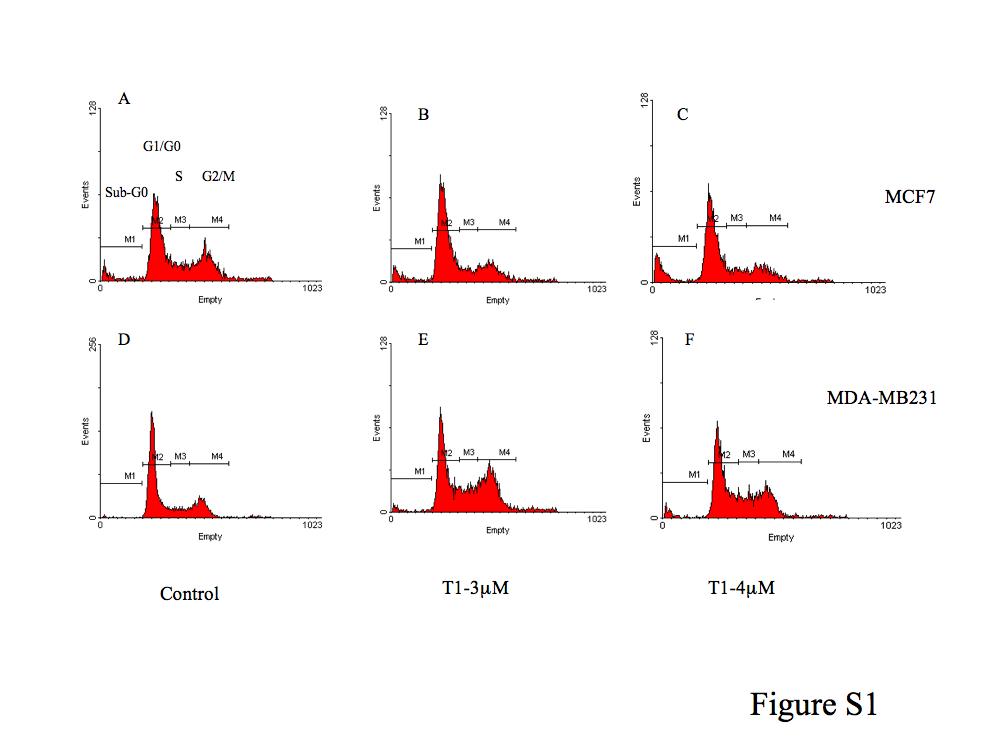

Supplement: Figure S1 — Representative FACS histograms showing the effects of T1 treatments (3 and 4µM) on cell cycle progression in MCF-7 (A-C) and MDA-MB231 (D-F) cell lines. (TIF) [file pone.0033656.s001.tif]

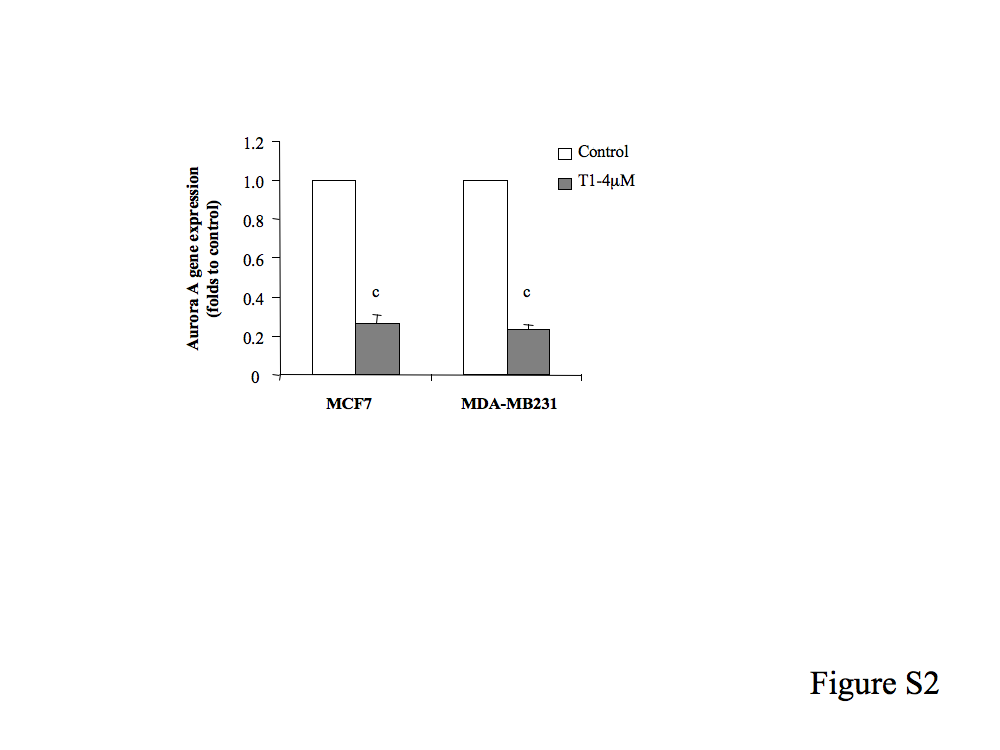

Supplement: Figure S2 — Effects of T1 treatment (4µM) on Aurora A gene expression in MCF-7 cells. Values were mean±SEM of three independent experiments in triplicates. The value with a letter is significantly different from that of the corresponding control (c, p<0.001). (TIF) [file pone.0033656.s002.tif]

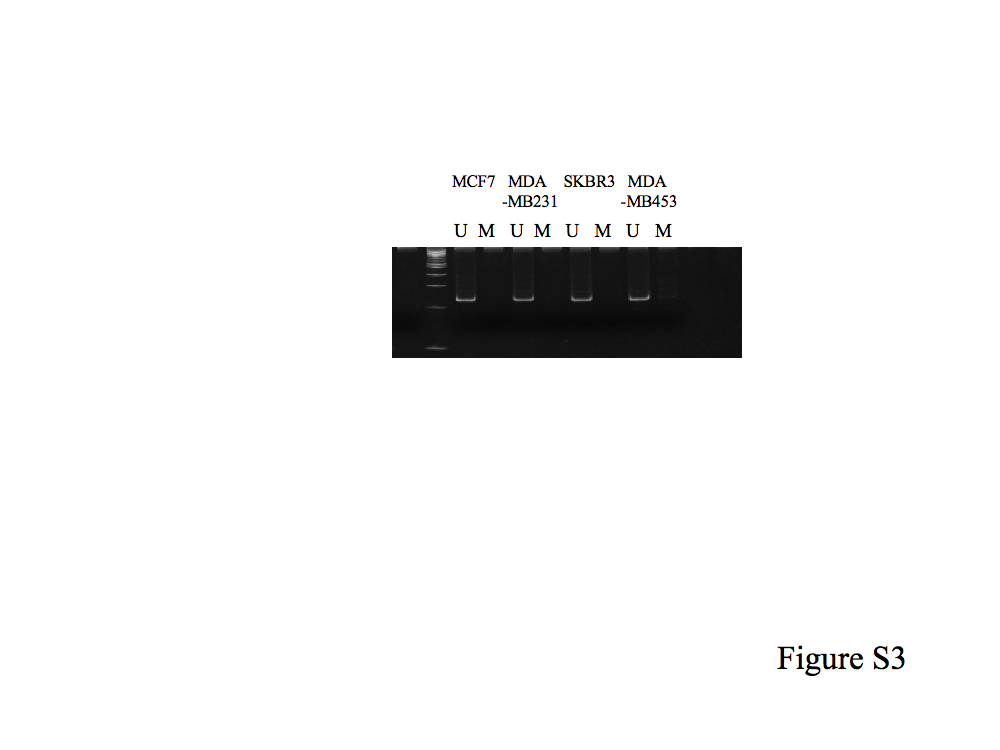

Supplement: Figure S3 — The representative image showing unmethylation status of Aurora A gene DNA promoter in MCF-7, MDA-MB231, SKBR3 and MDA-MB453 human breast cancer cell lines, as determined by methylation specific PCR (MSP). (TIF) [file pone.0033656.s003.tif]
