# Supplementary material for: Tanshinones Inhibit the Growth of Breast Cancer Cells through Epigenetic Modification of Aurora A Expression and Function
Source: PLoS One. 2012 Apr 2;7(4):e33656. doi: 10.1371/journal.pone.0033656 (PMC3317444; doi:10.1371/journal.pone.0033656)
Supplement: Table S1 — Sequences of the primers. (DOC) [file pone.0033656.s004.doc]

Table S1. Sequences of the primers

| β-actin for RT-PCR(F) | 5’-GATGAGATTGGCATGGCTTT-3 |
| --- | --- |
| β-actin for RT-PCR(R) | 5’-CACCTTCACCGTTCCAGTTT-3’ |
| Survivin for RT-PCR(F) | 5’-GCCCAGTGTTTCTTCTGCTT-3’ |
| Survivin for RT-PCR(R) | 5’-TCTCCGCAGTTTCCTCAAAT-3’ |
| Aurora A for RT-PCR(F) | 5’-CATCTTCCAGGAGGACCACT-3’ |
| Aurora A for RT-PCR(R) | 5’-CAAAGAACTCCAAGGCTCCA-3’ |
| Unmethylation primer for survivin (F) | 5’-GGTGGGAGGATTATAATTTTTG-3’ |
| Unmethylation primer for survivin (R) | 5’-CCACCACCACCACCTCTAC-3’ |
| Methylation primer for survivin (F) | 5’-GGCGGGAGGATTATAATTTTCG-3’ |
| Methylation primer for survivin (R) | 5’-CCGCCACCTCTACCAACG-3’ |
| Unmethylation primer for Aurora A (F) | 5’-TTGAGTTTGTATTTTTGTTGGTTTG-3’ |
| Unmethylation primer for Aurora A (R) | 5’-AAACCCAAAAAACAACAATTTCCA-3’ |
| Methylation primer for Aurora A (F) | 5’-AGTTCGTATTTTCGTCGGTTCG-3’ |
| Methylation primer for Aurora A (R) | 5’-GAACCCGAAAAACGACGATTTCCG-3’ |
| Promoter primer for survivin (F) | 5’-CGCTGGGTGCACCGCGACCAC-3’ |
| Promoter primer for survivin (R) | 5’-CGGCCTTCTGGGAGTAGAGGCG-3’ |
| Promoter primer1 for Aurora A (F) | 5’- TTGGAAGACTTGGGTCCTTG-3’ |
| Promoter primer1 for Aurora A (R) | 5’-ACACTCAGGGACGGAGGAAC-3’ |
| Promoter primer2 for Aurora A (F) | 5’-CTCTTGCTTTTCTAAGAAC-3’ |
| Promoter primer2 for Aurora A (R) | 5’-CGCACTTGCTCCCTAAGAAC-3’ |
| Promoter primer3 for Aurora A (F) | 5’-CTCGCCAGGTAAACAGAAGC-3’ |
| Promoter primer3 for Aurora A (R) | 5’-CCTTAGTTCGCCTCTGCATC-3’ |
| Promoter primer4 for Aurora A (F) | 5’-GGTTCAAGGAGGTCAGGACA-3’ |
| Promoter primer4 for Aurora A (R) | 5’-GGTGCCCTCAGTTCAAGGTA-3’ |
| Promoter primer5 for Aurora A (F) | 5’-TTGATGAGATGCAGGAGCTG-3’ |
| Promoter primer5 for Aurora A (R) | 5’-ACAGAACCAAAGTGGGCTTG-3’ |
