# Supplementary material for: Tanshinones Inhibit the Growth of Breast Cancer Cells through Epigenetic Modification of Aurora A Expression and Function
Source: PLoS One. 2012 Apr 2;7(4):e33656. doi: 10.1371/journal.pone.0033656 (PMC3317444; doi:10.1371/journal.pone.0033656)
Supplement: Table S2 — Raw Ct values for Figure 7 A, B, and C. (DOC) [file pone.0033656.s005.doc]

Table S2. Raw Ct values for Figure 7 A, B, and C

Figure 7A: Aurora A gene expression

| Group | Control | 5-AZA | SB |
| --- | --- | --- | --- |
| Ct of Aurora A | 29.401±0.07 | 29.38±0.08 | 28.62±0.23 |
| Ct of β-actin | 18.73±0.17 | 18.38±0.21 | 19.79±0.27 |

Figure 7B: Aurora A H3 acetylation

| Cell line | HMEC | | MCF-7 | |
| --- | --- | --- | --- | --- |
| Promoter | Primer4 | Primer5 | Primer4 | Primer5 |
| Ct of sample | 36.24±0.35 | 37.41±0.53 | 34.89±0.06 | 34.23±0.31 |
| Ct of Input control | 32.61±0.37 | 31.61±0.46 | 32.09±0.32 | 31.57±0.39 |

Figure 7C: Aurora A H3 acetylation

| Experimental groups | Control | | T1 treatment | |
| --- | --- | --- | --- | --- |
| Promoter | Primer4 | Primer5 | Primer4 | Primer5 |
| Ct of sample | 37.27±0.57 | 38.19±1.44 | 38.99±0.91 | 39.15±1.85 |
| Ct of Input control | 32.98±1.16 | 32.34±1.19 | 34.03±1.26 | 33.34±1.26 |

Values are means ± SEM of three independent experiments in triplicates.
